# *In situ* and low-cost monitoring of particles falling from freshwater animals: from microplastics to parasites

**DOI:** 10.1093/conphys/coaa088

**Published:** 2020-09-26

**Authors:** Karel Douda, Felipe Escobar-Calderón, Barbora Vodáková, Pavel Horký, Ondřej Slavík, Ronaldo Sousa

**Affiliations:** 1 Department of Zoology and Fisheries, Czech University of Life Sciences Prague, Kamýcká 129, CZ-165 00, Prague, Czech Republic; 2 CBMA, Centre of Molecular and Environmental Biology, Department of Biology, University of Minho, Campus Gualtar, 4710-057 Braga, Portugal

**Keywords:** Aquatic animals, drop-off, fish, freshwater mussels, glochidia, host–parasite relationships, microparticles, microplastics

## Abstract

A simple and low-cost method of monitoring and collecting particulate matter detaching from (or interacting with) aquatic animals is described using a novel device based on an airlift pump principle applied to floating cages. The efficiency of the technique in particle collection is demonstrated using polyethylene microspheres interacting with a cyprinid fish (*Carassius carassius*) and a temporarily parasitic stage (glochidia) of an endangered freshwater mussel (*Margaritifera margaritifera*) dropping from experimentally infested host fish (*Salmo trutta*). The technique enables the monitoring of temporal dynamics of particle detachment and their continuous collection both in the laboratory and *in situ*, allowing the experimental animals to be kept under natural water quality regimes and reducing the need for handling and transport. The technique can improve the representativeness of current experimental methods used in the fields of environmental parasitology, animal feeding ecology and microplastic pathway studies in aquatic environments. In particular, it makes it accessible to study the physiological compatibility of glochidia and their hosts, which is an essential but understudied autecological feature in mussel conservation programs worldwide. Field placement of the technique can also aid in outreach programs with pay-offs in the increase of scientific literacy of citizens concerning neglected issues such as the importance of fish hosts for the conservation of freshwater mussels.

## Teaser

A new technique using a field-deployable device enables capturing of particles (such as parasite stages and microplastics) dropping from (or interacting with) aquatic animals to analyze their dynamics experimentally.

## Introduction

The inherent complexity of various ecological processes warrants the efficient combination of laboratory and field experiments. However, despite the rapid development of tools designed to enhance field data collection (e.g. remote electronic control systems; Burnett *et al*., 2013; Wilson *et al*., 2014; Kubizňák *et al*., 2019) there still exist many research areas where no field-based, low-cost technical solutions are available for primary data collection. This situation is especially true for aquatic organisms, which may restrict the collection of data for many ecological and physiological processes to laboratories or during short-term invasive sampling campaigns (e.g. Barber *et al*., 2008; Hart *et al*., 2018).

Diverse research fields such as ecological parasitology, ecotoxicology, aquatic animal nutrition and reproductive biology require techniques to collect objects detaching from live aquatic animals. Laboratory methods exist for collecting parasite stages (Dodd *et al*., 2005; Marchiori *et al*., 2013), faecal pellets (Shomorin *et al*., 2019) or eggs (Gonsar *et al*., 2012) in recirculating systems on screens. These methods make the following possible: the study of the time course of particle detachment at the individual level, the evaluation of the daily feed intake of animals in aquaculture facilities and the collection of particles over extended periods of time. The collection of fallen particles has also proven essential for understanding various aspects of aquatic animal physiology such as digestibility analyses (Da Mota *et al*., 2015; Dvergedal *et al*., 2019) and host–parasite compatibility (e.g. Rogers-Lowery *et al.*, 2007; Dodd *et al.*, 2005; Donrovich *et al.*, 2017). However, laboratory approaches have the disadvantage of being limited to a range of model organisms for which long-term holding under artificial conditions has been mastered (Levy and Currie, 2015; Russell *et al*., 2017). Consequently, a lack of data persists for most animal species in which the laboratory approach is not feasible because it can inadvertently affect their behaviour, biological rhythms and physiology (Calisi and Bentley, 2009). This, coupled with the high operational costs and labor requirements of the research facilities needed, makes the laboratory approach unsuitable in many areas of ecology and conservation physiology research.

Here, and as a proof of concept, we describe a new technique that can be used in the field to collect objects detaching from (or interacting with) aquatic organisms using a flow-through cage system. For this, we assessed the technique’s efficiency to collect (i) microplastics (polyethylene microspheres) and (ii) juveniles (post-parasitic stage) of an endangered species. We choose these two cases because in one hand microplastics have gain traction as a recent relevant research topic due to the possible deleterious effects on consumption, growth, reproduction and survival of aquatic animals (Foley *et al*., 2018). However, the level of knowledge in freshwater ecosystems lags behind what has been explored in marine ecosystems (Eerkes-Medrano *et al*., 2015), and the interaction between microplastics and freshwater organisms is particularly understudied for wildlife compared to laboratory models (de Sá *et al*., 2018). On the other hand, and given their complex life cycle, we used one species of freshwater mussels (Bivalvia: Unionida), one of the most threatened faunal groups in the planet, which in the past decades has been highly studied and subjected to several conservation management plans including captive breeding programs (Lopes-Lima *et al*., 2017; Ferreira-Rodríguez *et al*., 2019). This group of bivalves has a temporarily parasitic larval stage (glochidium; size, 50–400 μm) that must attach to the body surface of a suitable fish and become encapsulated in the epithelial layer to metamorphose into a juvenile mussel (Kat, 1984; Modesto *et al*., 2018), then it ruptures the capsule and detaches from the host. Freshwater mussel–fish relationships have become useful models for addressing questions in fish ecology (Gopko *et al*., 2018; Horký *et al*., 2019; Methling *et al*., 2019), toxicology (Defo *et al*., 2019; Douda *et al*., 2019) and the conservation biology of host–affiliate relationships (Tremblay *et al*., 2016; Schneider *et al*., 2017).

Various laboratory methods have been established for the study of the metamorphosis success rate of glochidia using adapted multi-unit laboratory fish-holding recirculation systems (Dodd *et al*., 2005; Hazelton *et al*., 2013; Douda *et al*., 2018; Dudding *et al*., 2019), sets of aquaria adapted for periodical or continuous siphoning (Reis *et al*., 2014; Douda *et al*., 2014; Reichard *et al*., 2015; Donrovich *et al*., 2017) or other custom-made fish holding tanks (Taeubert *et al*., 2013; Eybe *et al*., 2015; Huber and Geist, 2017; Soler *et al*., 2018). However, some of these methods can be problematic (especially when used for fish collected in the field), leading often to high fish mortality during experiments (Taeubert *et al*., 2013; Huber and Geist, 2017; Soler *et al*., 2018), reducing the representativeness of the results. The fact that there is currently no available method for the collection of mussel juveniles falling from the fish host under field conditions strongly limits our ability to test new potential hosts in species where transport to the laboratory is problematic, or in areas without suitable laboratory infrastructure. Such limitation is one of the main reasons for the insufficient knowledge of the host sources of freshwater mussels (Modesto *et al.*, 2018) and for the need to look for new methods that are feasible without a laboratory (Hart *et al.*, 2018).

Given the above-described background and the need to develop simple methods that increase information about basic autecological processes, the main aim of this study was to describe a low-cost technique that may be employed in several ecological topics related to conservation physiology of aquatic animals (from simple assessment of animal-microplastics interactions to more complicated analysis of host–parasite relationships). We also discussed the use of this technique in other topics, including outreach programs.

## Methods

To demonstrate the utility of the technique in real-world ecological problems, we present two examples that can be performed with this device, whether it is in a laboratory or a field. The first quantifies the interaction time and capture efficiency of the device for externally added standard particles in the laboratory with potential use in the study of animal–microplastics interactions. The second illustrates a breakthrough advance in field-based fish–glochidia interaction studies by addressing questions previously tractable only under laboratory conditions.

## Principle and construction of the device

### Floating board and cages

**Figure 1:**
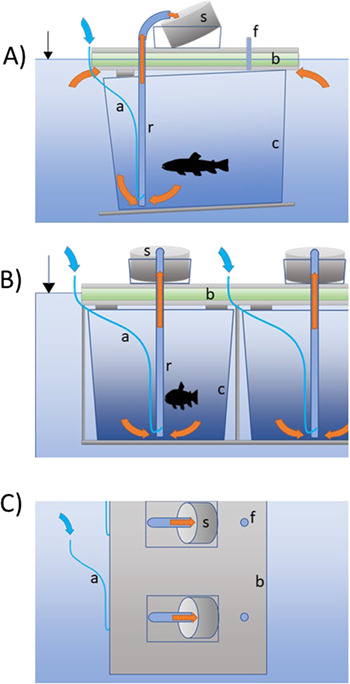
Side (A), front (B) and top (C) schematic view of FDPC. r, riser pipe; s, filter cylinder; f, feeding and calibration port; b, floating board; c, polypropylene cage; a, air delivery hose; red arrows, water flow; blue arrow, air flow.

**Figure 2:**
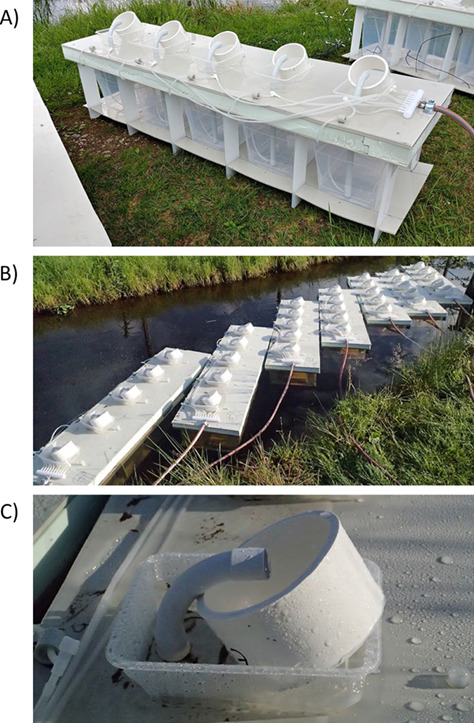
Example use of the FDPCs for the sampling of *Margaritifera margaritifera* juveniles dropping from host fish (*Salmo trutta*): (A) polypropylene structure arrangement for a 5-cage system, (B) field deployment of 7 systems with 34 x 22 x 28 cm cages and (C) detail of the collection cylinder.

The floating drop-off particle collector (FDPC) unit operates on a free-floating board (width: 50 mm; polystyrene) weighed down from the upper and bottom side by protective sheets (thickness: 5–10 mm; polypropylene). Five animal holding tanks are suspended below the floating board, each positioned within five different divisions (Figs 1 and 2). The divisions are created by heat welding 5–10 mm polypropylene sheets perpendicular to the main floating board at regular intervals. The bottom of the tank lies on a single sheet (thickness: 5 mm) to which the perpendicular sheets of the divisions are heat welded. In each division, an experimental tank is placed to form a cage for the fish. Commercially available boxes with a smooth and undiversified internal surface can be used. Here, polypropylene fish tanks (volume: 20 L, length x width x height: 34 x 22 x 28 cm; T-Box S, Keter Italia S.p.A., Italy) were used. To firmly fit the tanks into each division and allow passage of water from the exterior, a gap between the floating board and the tanks in each division was created by inserting two silicone blocks (height: 12 mm) with smooth edges to prevent injury to the fish. The dimensions of the silicone blocks need to be adjusted to the size of the organisms tested.

### Air and water flow

The FDPC device operates using the principle of airlift pumping. Each tank is equipped with its own riser pipe (diameter: 20 mm; PVC pipe), the pressured air required by the units during operation is provided by land-positioned compressors. The air is injected into the bottom part of the riser pipes in each holding tank, and because the mixture of air and water is less dense than the surrounding water, it rises to the top aperture, sucking water and solids from the bottom of the tank and transporting them to the collection net positioned above the main floating board. The riser pipe outlet in the top of the FDPC is connected to a 90-degree bend, ending 130 mm above the water surface level (80 mm above the floating board surface), just above a collecting filter cylinder. The main air supply line starts with an electrical air compressor to which a hose (inner diameter: 135 mm) is connected. The other end of the hose is attached to one end of the FDPC device on top of the floating board. From there, a manifold air divider valve distributes the air to the different riser pipes (or is left open to stabilize the airflow if needed—see below) through 4 mm (inner diameter) silicone tubes. Each tube is equipped with a two-way air control valve. A single air compressor can feed several FDPC units; here, one 100-W compressor (airflow: 110 L min^−1^; air pressure: 0.035 MPa, 102 W; Hailea ACO-009, China) was successfully used to feed 2–3 FDPC units.

Because the flow rate determines the entrapping effectivity of the pump, it is necessary to measure the water flow through each filter and standardize it among tanks. The water flow through the outlets can be measured by a graduated collection vessel placed where the filter cylinders are usually located and adjusted by changing the amount of air being pumped to the riser pipes using the two-way air valves connected to the air tubes for each tank. The mean ± SD water flow through the individual tanks under the above-described settings during both experiments was 45.6 ± 9.6 mL s^−1^.

### Collecting cylinders

The collecting filter cylinders (Fig. 2C) are made from PVC pipe (diameter: 115 mm; height: 65 mm) with a nylon screen of specific mesh adjusted to the size of the monitored particles attached (here, we used a loop size of 139 μm). The filter cylinders are placed into PVC positioning box fixed on top of the FDPC, which stabilizes the position of the filter at the desired angle against the riser pipe outlet (we used 45 degrees as the optimal angle). The height of the openings in the positioning box determines the water level around the cylinder and allows the presence of a pool of water above the bottom part of the screen. This pool keeps the particles under water after recovery if needed. Alternatively, other type of screens, such as wedge wire screens, can be used, if necessary, to keep the recovered material out of the water (not tested here, see Shomorin *et al*., 2019 for details).

### Feeding and calibration port

The FDPC is equipped with a set of additional ports located in each section opposite to the main riser pipe. A silicone tube (inner diameter: 10 mm) is positioned in each opening (ending 5 mm above the floating board). The function of these apertures (hereinafter feeding and calibration ports) is to allow the introduction of external food items during the experiment (if needed) or a known number of particles of interest for exposure or calibration purposes.

The cost of the system described for all experiments was approximately $1105 per 35 tanks distributed among 7 FDPC units (see Table S1 for a detailed description). The system can be easily built using an electric saw, plastic welding heat gun (with compatible polypropylene rods), electric screwdriver and drill bit and moved to any water body with available electricity on the bank. While we used plug-in compressors and a 230-volt power connection, solar or battery sources alongside voltage converters can be used to make the system more portable. The device does not require any construction of solid structures or racks and adapts to possible fluctuations in water level.

## Proof of concept

### Example 1: polyethylene microspheres

Cyprinid fish *Carassius carassius* (Linnaeus, 1758) individuals (mean total length: 127 mm; mean body mass: 34 g) obtained from a laboratory breeding population at the Czech University of Life Sciences Prague (Czech Republic) were kept in a 250-L aquarium at 15°C, and a light–dark regime of 12:12 h before the start of the experiment. Fish were fed daily with commercial fish pellets (Pond Pellet, 5–6 mm; Tetra, Germany) before and during the experiment. A FDPC unit was installed in a 200 x 100 x 100 cm (length x width x height) laboratory tank with dechlorinated tap water (1800 L) under identical temperature and photoperiod conditions as described above. On the day of the start of the experiment, five randomly selected fish were extracted from the aquarium and placed into each of the tanks of the FDPC.

The microplastics, Red Polyethylene Microspheres (1.12 g cc^−1^, 500–600 μm), were purchased from Cospheric (Santa Barbara, CA, USA). To prevent the particles from floating or creating clumps, an organic food-grade surfactant (Tween 80 Biocompatible Surfactant, Cospheric, CA, USA) was used. The microplastics (106–114 particles) were introduced into the respective tanks in the FDPC with the help of a syringe attached to a silicone tube. The assessment of flushed particles was performed at 1, 6, 24, 48, 72, 96, 120, 144 and 168 h after the start of the experiment. At each time, the five collecting cylinders of the FDPC were replaced with new clean filters, and the used filters were then observed under a microscope to assess the number and status of particles recovered.

### Example 2: parasitism success in an endangered species

A second experiment applied the FDPCs to monitor parasitism success and to collect juveniles of the freshwater mussel *Margaritifera margaritifera* (Linnaeus, 1758) detaching from its fish host, *Salmo trutta* Linnaeus, 1758. It should be noted that previous field studies have been restricted to evaluation of glochidia attachment intensity observed on wild fish (Salonen *et al.*, 2017; Dias *et al.*, 2020), whereas evaluation of metamorphosis success has been limited to laboratory studies (e.g. Douda *et al*., 2017; Schneider *et al*., 2017). The success of *M. margaritifera* parasitization was tested using larvae from two different source populations (with different qualities of glochidia) experimentally infesting host fish from two different populations.

For this, the experimental *S. trutta* were caught by electrofishing (650 V, 4 A, pulsed D.C.) in two streams (population Fish-A, Živný potok stream, 49°2′39′′N, 14°1′32″E; population Fish-B, Častá stream, 48°55′4′′N, 13°40′27″E) within the Vltava River basin (Czech Republic) with no current *M. margaritifera* populations. The fish were anaesthetized with 2-phenoxy-ethanol (0.2 mL L^−1^; Merck KGaA, Germany), measured (total length: mean 164 mm, range 95–216 mm), weighed (body mass: mean 40 g, range 6–90 g) and individually marked 6–13 days before the infestations. Passive integrated transponders (PITs; Trovan ID100, 0.1 g in air, 12 × 2.1 mm; EID Aalten B.V., Aalten, the Netherlands) were inserted into the dorsal muscle using a syringe. After marking, the fish were kept in side-arm of the Vltavský potok stream (48°59′0.5”N, 13°39′38″E) in the Šumava National Park hatchery before infestation with glochidia.

Parasitic glochidia of *M. margaritifera* were obtained from female mussels sampled from two different populations in the Vltava River basin (Czech Republic) (population Gloch-A, Blanice River, 48°55′34′′N, 13°58′12″E; population Gloch-B, Malše River, 48°39′01.5′′N 14°28′00.3″ E). To obtain glochidia of *M. margaritifera*, several mussel individuals were monitored in the field and when glochidia release was observed, the individuals were collected and placed into a shallow 5-L vessel to stimulate further glochidia release. The clumps of glochidia released were extracted with the help of a pipette and observed under a microscope to assess viability. Then, the glochidia were transferred to 5-L containers with river water. Two separate mixtures of glochidia obtained from 35 and 3 female mussels from populations Gloch-A and Gloch-B, respectively, was used (August 2018, 7 days between the two infestation events). After the glochidia were extracted, the females were returned to the same collection location. The containers with glochidia were transported immediately in cooling boxes to the Vltavský potok stream, where the infestations were performed in the same day.

Fish were infested with glochidia in August 2018 in a common bath suspension with densities of 15 400 ± 3666 and 11 200 ± 3516 (mean ± SD) glochidia L^−1^ for populations Gloch-A and Gloch-B, respectively. Density was assessed by counting ten 1-mL subsamples. The viability of the glochidia was tested by evaluating their snapping action in a NaCl solution immediately before infestation (Roberts and Barnhart, 1999). The average percentage of viable (reacting) glochidia in the inoculation bath was 31% in Gloch-A and 74% in Gloch-B. The infestation procedure lasted 15 min, and the density of fish in the glochidia suspension was 1 fish L^−1^. Individuals from both fish populations were infested in a common bath. The control (uninfested) fish were treated with the same handling procedures (i.e. transfer between baths). After infection, the fish were released into a seminatural side-arm of the Vltavský potok stream with a natural gravel/sand bottom (length: 47 m; width: 2–3 m; depth: 0.1–0.6 m) and an adjacent earth pond (area: 139 m^2^; max. depth: 1.5 m).

The monitoring of falling juvenile mussels using FDPCs was initiated upon reaching the sum of temperatures reported as usual for the start of juvenile mussels dropping from host fish (Hruška, 1992), which occurred in June 2019 (total number of days from infestation: Gloch-A, 310 and Gloch-B, 317). The average daily temperature during the whole period ranged between 0.2 and 16.1°C, and the total sum of daily degrees until placement in the FDPCs ranged between 1573 and 1783. Seven FDPC units (total: 35 holding tanks) were placed directly at the site where the fish had spent the previous part of the parasitic period. The fish were caught as described above and were gradually placed in the FDPCs, where they spent 6–8 days at average daily temperature during monitoring 13.2 ± 1.0°C (range: 12.0–15.1°C). The relative body weights (condition factors) of 12 randomly selected fish individuals were determined using the equation K = 100 x somatic weight (g)/(standard length [cm])^3^ before the placement and after the removal of the FDPCs. We have verified the functionality of the feeding and calibration ports for live feeds but did not add food items on a regular basis because the presence of live aquatic invertebrates (mayfly larvae, benthic crustaceans) was regularly detected on the filters, indicating natural food being supplied to the tanks in this experiment. The FDPC collecting cylinders were exchanged at 1–2-day intervals and inspected at 10–40x magnification under the microscope. Juvenile mussels falling from the hosts were classified as live if valve or foot movement was observed. The average rate of parasite detachment from fish (number of juvenile mussels day^−1^ g^−1^ of fish body weight) was determined together with the success rate of metamorphosis during the monitored period (the percentage of dead and live juveniles falling from the fish). Fish individuals were returned to their site of capture after the experiment.

To verify the temperature conditions in the FDPCs, a datalogger (temperature accuracy: 0.1°C; Hobo, Onset, USA) was placed inside and outside the device, recording data every 15 min for 7 days. For the field flushing efficiency test, uninfected control fish were placed in 3 tanks of an FDPC, and 36–74 mussel juveniles were then placed inside the unit using the feeding port. For the next 96 hours, monitoring was performed as described above to determine the success of recapture.

We used paired Wilcoxon rank-sum tests to determine whether the detachment rate of juveniles and metamorphosis success (arcsine-transformed proportion of viable juveniles) differed between the different host–parasite population combinations. Paired t-tests were used to compare fish condition factor and temperature differences. All analyses were performed in R 3.5.2 (R Core Team, 2019).

## Results and discussion

### Example 1: polyethylene microspheres

Mortality of *C. carassius* during the experiment was zero and there were no signs of skin or fin injuries. The capture efficiency using polyethylene microspheres showed a mean (±SD) particle flushing efficiency of 95.9 ± 4.5%. Most of the particles (91.3 ± 5.1%) were flushed in the first 24 h, and the last particles were recovered 120–144 h after insertion (see Table S2 for details). Microspheres recovered in the later stages of the experiment (72–144 h) showed that they had been mechanically damaged and small particle fragments were also present. Although it was not specifically studied here, the relatively long residence time and physical damage of these particles indicated that they had passed through the digestive tract of the fish and were harmed by the pharyngeal teeth.

By capturing particles leaving the enclosure space, the device allows determining the time and concentration of exposure to particles while being held under ambient environmental conditions. The availability of well-defined (colour, size, relative density, shape) plastic particles for experimental purposes enables this to be done effectively and offers new experimental possibilities. In addition, water flow through the system can be regulated to adjust the residence time.

### Example 2: parasitism success in an endangered species

Host fish (*S. trutta*) mortality was zero, there were no signs of skin or fin injuries, and the condition factor of fish did not change (*P* > 0.05) during the experiment. The flushing efficiency of the *M. margaritifera* juveniles in the field (recapture rate of added juveniles) ranged between 88.1% and 100.0%, and 90.4% to 98.6% of juveniles were recovered within the first 24 hours. There was no difference in the temperature recorded inside and outside the devices (*P* > 0.05; mean difference ± SD: 0.05 ± 0.08°C).

**Figure 3:**
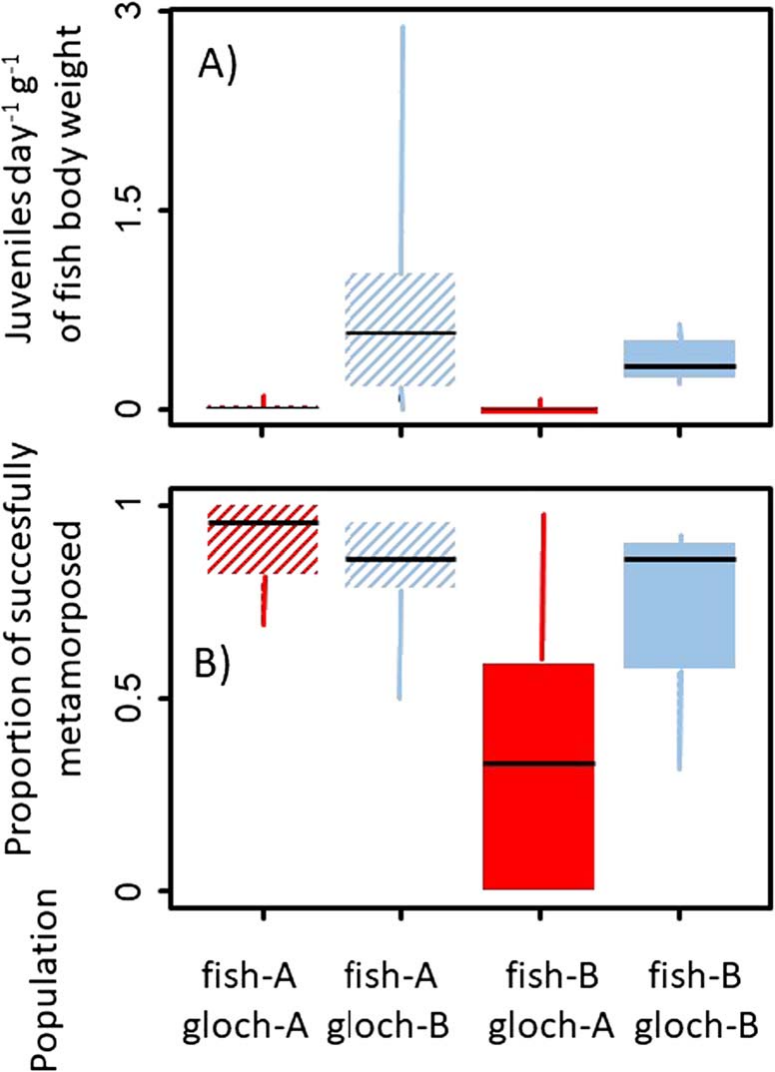
(A) The rate of *Margaritifera margaritifera* juvenile detachment per gram of fish body weight (pairwise Wilcoxon test, differences between mussel populations—*P* < 0.001, *n* = 4–18) and (B) the proportion of successfully metamorphosed glochidia during the 14-day monitoring period (1573–1783 degree days from infestation; pairwise Wilcoxon test, all *P* > 0.5, *n* = 4–18) as detected by the FDPC. The median, interquartile range and min/max for different combinations of source populations of parasites (red/blue) and hosts (hatched/unhatched) are displayed.

The estimated average *M. margaritifera* juvenile detachment rate across all fish was 0.16 ± 0.47 juveniles day^−1^ g^−1^, and the average percentage of successfully metamorphosed glochidia was 74.0 ± 30.2%. In terms of the detachment rate, there were significant differences between the fish infested with different mussel populations (Fig. 3A). Fishes infested with Gloch-A had a significantly (*P* < 0.001) lower juvenile detachment rate (0.01 ± 0.02 juveniles day^−1^ g^−1^) than fish infested with Gloch-B (0.68 ± 0.79 juveniles day^−1^ g^−1^); but there were no detectable differences in the juvenile detachment rate between host fish populations (*P* > 0.05).

In terms of juvenile mussel metamorphosis success, a slightly higher percentage of live juveniles was associated with the fish infested with Gloch-B (78.6%, versus 70.4% for Gloch-A, Fig. 3B), which corresponds with the higher detachment rate in this fish population, but no significant differences were detected (all *P* > 0.05). A total of 2377 detached *M. margaritifera* juveniles were sampled.

These results show that FDPC is able to detect differences in the physiological compatibility of different combinations of source glochidia and host populations. In our case, the results demonstrate a greater efficiency in the use of *S. trutta* hosts by the glochidia from population B possibly due to immunological mechanisms (Rogers-Lowery *et al*., 2007), or due to a lower quality of glochidia produced by population A (indicated also by the initial viability analysis, see above), a common problem in freshwater mussel propagation activities (Patterson *et al*., 2018). In terms of conservation application, it shows us which mussel population provides a more efficient source of glochidia for possible rescue or bioindication breeding. On the other hand, the results do not indicate a different ability of the two fish strains to host *M. margaritifera* due to local adaptation as recorded by previous studies (e.g. Douda *et al*., 2017; Schneider *et al*., 2017). Although a more complex study design would be needed to take into account the effect of glochidia viability, and test the effects of recorded lower metamorphosis success rates in the combination of Fish-B and Gloch-A populations, both populations can be considered physiologically compatible hosts. Therefore, the FDPC allows addressing the geometry of local adaptations between mussels and fish by studying metamorphosis success directly in the field (in remote geographical locations, under natural temperature and photoperiod regimes and water quality conditions), which to our knowledge has not been possible before.

### General discussion and way forward

This study described the construction of a field-deployable floating device for the continuous monitoring of detachment or interaction regimes of particles associated with aquatic animals. This novel approach is cheap and mobile, and can be used in other type of environmental studies (e.g. faeces-based molecular diet analyses and ingested microplastic quantification) (Nelms *et al*., 2019) using fishes and other aquatic animals (e.g. crayfish and other macroinvertebrates, amphibians).

The use of nonlethal methods to collect fish faeces from animals exposed to microplastics can prove to be a valuable addition to this type of study (Hoang and Felix-Kim, 2019; Kazour *et al*., 2018), allowing us to record the dynamics of microplastic excretion. The device can be especially useful when a long transport distance would be necessary and risky, or when the acclimatization to the available laboratory conditions is problematic (Calisi and Bentley, 2009). It should be highlighted that this method cannot be easily used for non-specific monitoring of plastics in the field due to their possible source from the surrounding environment and the device itself (Löder and Gerdts, 2015; Li *et al*., 2018). On the contrary, the proposed use tested here as proof of concept consists on the controlled exposure of organisms to plastics of specific properties and detectability (Shim *et al.*, 2017; Heinrich *et al.*, 2020) either before or during (as showed here) the placement into the system and monitoring the regime of particles-animal interaction under natural water quality and temperature and photoperiod regimes.

Although we showed that the FDPC is ideal for collecting particles dropping from or interacting with fish in laboratory and oligotrophic habitats, slight alterations to the presented system can further increase its range of applications. The use of wedge screens can be suitable for the collection of faeces more effectively (Dvergedal *et al*., 2019; Shomorin *et al*., 2019), and the system can be surrounded by protective nets to prevent input of other prey items (when providing food items manually) or other types of potential interference (e.g. in the case of filter-feeders). Another possibility to extend the usability of the system is the implementation of technological accessories to record and report online the behavioural activity of the objects studied, environmental conditions and system malfunctions, which has not been possible without continuous operator presence until recently (Kubizňák *et al.*, 2019; Sheehan *et al.*, 2020).

Another promising opportunity for the FDPC application is the conservation biology of freshwater mussels, which are declining worldwide (Lopes-Lima *et al*., 2014, 2018). The use of FDPCs in mussel conservation can involve two main activities. First, as demonstrated here using *M. margaritifera,* the FDPC represents a cheap, reliable, and deployable mean of testing the glochidia metamorphosis success rate—a critical knowledge for the determination of conservation units and host resource management (Modesto *et al*., 2018). Second, the FDPC can be a powerful tool for the recovery of both larvae and juveniles from endangered freshwater mussels. The use of this (or similar) techniques to increase our knowledge about basic autecological features of freshwater mussels is highly welcome, because it has been shown that adult mussels held in the laboratory conditions over long terms exhibit lower growth, altered metabolism and higher mortality (Patterson *et al.*, 2018; Roznere *et al.*, 2014). Although a great increase in the number of studies addressing ecological and conservation issues of freshwater mussels can be found in the past decades, the reality is that basic information on key autecological (e.g. distribution, density, population size structures) features are still lacking (Lopes-Lima *et al*., 2020) especially in some areas where equipped laboratories or personal are not available. In fact, one key information gap is their reproduction and the metamorphosis of glochidia to juveniles. The device and methodology described here can overcome some of the bias (water quality, feeding and temperature differences) already described in the usual laboratory procedures and can help to expand this type of research into new geographical areas.

The device also has good potential for use in other biotic interactions. For example, Trematoda parasites produce in their intermediate (molluscan) host free-living larvae (cercariae), which swim actively or float passively in the water to find and infect the next host. An important branch of aquatic parasitology is the estimation of cercarial production. This is challenging in field conditions, because so far, the only way to estimate cercariae production has been to place the mollusc in a container for a period of time to be able to count the larvae (e.g. Taskinen 1998). The FDPC system described here can be an important innovation in this type of research. In addition, the possibility of placing the system in a freely accessible (compared to a remote and quarantined laboratory) location in the field can be beneficial for educational purposes. In the case of our field site near the fish hatchery of Šumava National Park, there were many opportunities to demonstrate the device to students and other visitors and thus communicate the fish-mussel host-parasitic system and their importance for conservation research programs.

Despite the possible advantages, it is important to take into consideration that although the device can be located in a river or a lake, it is not a physically natural habitat but an enclosure. Thus, it brings an effective advantage in some fundamental parameters (temperature and light regimes, and water quality), but on the contrary, it does not allow a number of natural behaviours (e.g. movements of animals to foraging areas or an interaction with substrate). Therefore, in particular cases, it will be necessary to determine whether the caging can affect the studied parameter. In the same vein, and although the device can eliminate the need of organisms transport over long distances and reduce the risks of disease transfer to or from laboratories or among catchments, as a field-deployable device, the FDPC itself could contribute to the movement of diseases and species. Because of this, we strictly recommend that all parts of the FDPC in contact with water must be disinfected and allowed to dry completely before being transported to another location.

In conclusion, collecting particles dropping from aquatic animals directly in the field not only provides opportunities to greatly increase the volume and type of data that can be collected in environmental parasitology or animal feeding ecology, but also enables the acquisition of new types of data in emerging research fields, such as microplastic pathway studies. Further research is needed to test FDPCs in other water systems and in association with other research topics. This system has excellent prerequisites for interconnection with remote electronic monitoring systems. Continued technological advances will make field-deployed floating systems an increasingly viable and versatile option without needing a sophisticated laboratory for holding organisms originating in the wild with the associated long-distance transport. The simple and low-cost design, field accessibility and easy operation also allow its use in outreach programs, increasing the scientific literacy of citizens in very specific topics such as the importance of fish to conserve critically endangered freshwater mussels.

## Supplementary material

Supplementary material is available at Conservation Physiology online.

## Author contributions

K.D. conceived the idea and designed the hardware. F.E-C., B.V. and K.D. performed the calibration, laboratory and field experiments. P.H., O. S. and K.D. collected the fish hosts and deployed the field units. All authors provided critical feedback, participated in manuscript writing and approved the final manuscript.

## Funding

This work was supported by the Czech Science Foundation [19-05510S] and the European Regional Development Fund [CZ.02.1.01/0.0/0.0/16_019/0000845, CZ.05.4.27/0.0/0.0/15_009/0004620].

## Supplementary Material

Supplementary_information_FDPC_ConsPhysF_REV1_coaa088
