# Supplementary material for: In situ and low-cost monitoring of particles falling from freshwater animals: from microplastics to parasites
Source: Conserv Physiol. 2020 Sep 26;8(1):coaa088. doi: 10.1093/conphys/coaa088 (PMC7519624; doi:10.1093/conphys/coaa088)
Supplement: Supplementary_information_FDPC_ConsPhysF_REV1_coaa088 [file supplementary_information_fdpc_consphysf_rev1_coaa088.doc]

**SUPPLEMENTARY INFORMATION**

**Article: *In situ* and low-cost monitoring of particles falling from freshwater animals: from microplastics to parasites**

**Table S1. List of materials used to build 7 FDPC units system of 5 tanks each (35 tanks)**

| **Material** | **Manufacturer** | **Price for a 35-tank system (USD)** |
| --- | --- | --- |
| Polypropylene sheets, MEPOLEN (PP-H) Platte extr., natur, 2000 x 1000 x 5/10 mm | Mepla, Mělník, Czech Republic | 600 |
| Extruded polystyrene sheets, Isover Styrodur 3000 CS 1250 x 600 x 50 mm | ISOVER, SGCP CZ, Czech Republic | 55 |
| Polypropylene boxes, 20 L, T-Box S | Keter Italia S.p.A., Italy | 200 |
| Silicone (DRINKTEC VMQ, 10/14 mm and 4/6 mm) and PVC (12.5/17 mm) hoses | Hojdánek CZ, České Budějovice, Czech Republic | 70 |
| Air compressors, Hailea ACO-009 Electrical Magnetic Air Compressor Pump * | Hailea, China | 110 |
| PVC pipes (20 mm inner diameter) and 90 gr. knees |  | 30 |
| Stainless steel connecting material (screws) and polypropylene rods for heat welding** |  | 40 |
| **Total** |  | **1105** |

*The airlift pump does not contain any rotating or mechanical elements; therefore, the pumped contents are not destroyed during transport. Although, other technical solutions exist that can also avoid destruction of the particles during extraction, airlift pumps are ideal for field setups since they can be built at very low initial cost, require low maintenance, are easy to install, resistant to clogging, and the regulation of ﬂow rate can be easily achieved by changing the amount of air being pumped.

**Heat welding is a simple technique for the joining of semi-finished plastic materials using high temperature (~300°C) device, which is widely used in aquaculture.

Table S2. The efficiency of flushing - % of polyethylene microparticles (1.12 g/cc, 500-600 µm) detected at n hours after the start of the calibration experiment.

| Unit | Particles added | 1 hour | 6 h | 24 h | 48 h | 72 h | 96 h | 120 h | 144 h | 168 h |
| --- | --- | --- | --- | --- | --- | --- | --- | --- | --- | --- |
| A | 118 | 36.44% | 92.37% | 97.46% | 98.31% | 99.15% | 99.15% | 99.15% | 100.00% | 100.00% |
| B | 108 | 49.07% | 90.60% | 94.84% | 95.68% | 95.68% | 95.68% | 95.68% | 97.38% | 97.38% |
| C | 114 | 41.23% | 91.23% | 92.92% | 94.62% | 97.16% | 97.16% | 97.16% | 97.16% | 97.16% |
| D | 113 | 30.97% | 82.67% | 88.60% | 90.30% | 93.69% | 96.23% | 96.23% | 97.92% | 97.92% |
| E | 106 | 48.11% | 67.60% | 82.86% | 83.71% | 86.25% | 87.10% | 87.10% | 87.10% | 87.10% |
| Average | | 41.17% | 84.89% | 91.34% | 92.52% | 94.39% | 95.06% | 95.06% | 95.91% | 95.91% |
| SD | | 6.89% | 9.30% | 5.13% | 5.11% | 4.45% | 4.16% | 4.16% | 4.52% | 4.52% |
